# A Case Report of Congenital Non-spherocytic Hemolytic Anemia in a Patient from India

**DOI:** 10.7759/cureus.2478

**Published:** 2018-04-13

**Authors:** Ruhi Sonaye, Shaheen Sombans, Kamleshun Ramphul

**Affiliations:** 1 Bharati Vidyapeeth Deemed University Medical College and Hospital, Sangli, Maharashtra, IND; 2 Department of Pediatrics, Shanghai Xin Hua Hospital Affiliated to Shanghai Jiao Tong University School of Medicine, Shanghai, CHN

**Keywords:** congenital non-spherocytic hemolytic anemia, india, pyruvate kinase deficiency, hexokinase deficiency, pyrimidine 5’nucleotidase deficiency, homozygous glucose phosphate isomerase deficiency

## Abstract

Congenital non-spherocytic hemolytic anemia (CNSHA) is a rare autosomal recessive condition that presents as a congenital hemolytic anemia. The absence of vital enzymes required for glycolysis such as homozygous glucose phosphate isomerase (GPI) and red blood cell (RBC) nucleotide metabolism predisposes the RBCs to hemolysis. No spherocytosis is seen on peripheral smear as well as no signs of immune-mediated destruction of RBCs. We present a rare case of a previously healthy 21-year-old female patient with CNSHA from India.

## Introduction

Congenital non-spherocytic hemolytic anemia (CNSHA) is an autosomal recessive condition that leads to a deficiency of vital enzymes required for glycolysis and red blood cell (RBC) nucleotide metabolism. It presents as a congenital hemolytic anemia and patients also show signs of jaundice. No spherocytosis is observed on peripheral smear and laboratory findings also exclude hemoglobin abnormalities. We present a rare case of a patient with CNSHA.

## Case presentation

A previously healthy 21-year-old female patient born out of a non-consanguineous marriage presented at the emergency department of the Osmania General Hospital in India complaining vomiting and loose motion of unknown origin for five days. On physical examination, she had no other systemic abnormalities and she was admitted for further tests. During the stay, she gradually developed a petechial rash over both lower limbs and her platelet count was slightly lower than the normal range at 130,000 per microliter of blood. Vomiting and diarrhea subsided and she eventually developed a yellowish discoloration of the sclera and her urine turned dark yellow. Her total and direct bilirubin was 12.58 mg/dL and 9.8 mg/dL, respectively. Physical examination at that point showed that her liver was palpable at 3 cm below the costal margin and splenomegaly was also noted.

**Figure 1:**
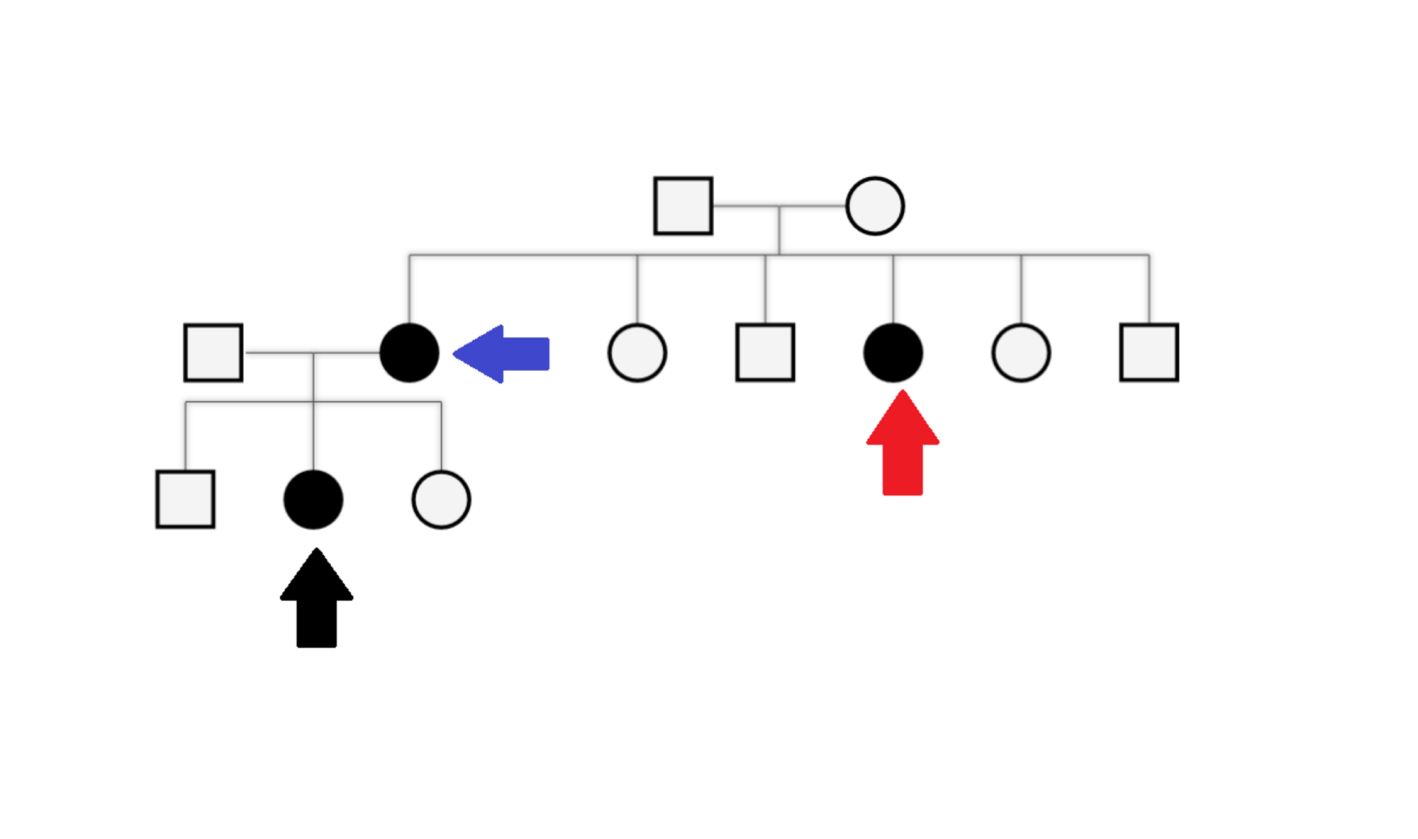
Pedigree diagram with the black arrow representing the patient, the blue arrow the patient's mother and the red arrow the patient's maternal aunt. All three of them had a history of similar symptoms.

Further laboratory testing showed that her percentage of RBCs was low (3.8 million cells/ul), mean corpuscular volume (MCV) and mean corpuscular hemoglobin (MCH) were normal at 98 fL/red cell and 30 picograms/cell respectively, while her mean corpuscular hemoglobin concentration (MCHC) was slightly in the lower range at 32 g/dL. Peripheral blood smear showed no spherocytes. Microcytes were present and hypochromic cells were also found. Osmotic fragility test showed normal osmotic fragility, while she also tested negative for Glucose 6 phosphate dehydrogenase deficiency. Hemoglobin electrophoresis was normal and the patient had a negative direct Coomb’s test and Sickling test. Ultrasound of the abdomen confirmed hepatosplenomegaly. A pedigree diagram was made (Figure 1) and it showed that her mother and maternal aunt had similar symptoms but were never diagnosed for the condition. Due to the lack of appropriate laboratory facilities, the enzyme levels were not tested and the physicians relied more on the clinical presentation, physical examination and other laboratory results to make a clinical diagnosis of congenital non-spherocytic hemolytic anemia. She was treated for anemia (hemoglobin level of 6.7 g/dL) with blood transfusions and additional symptomatic care such as proper hydration was initiated. The patient gradually improved over a week and was discharged.

## Discussion

Congenital non-spherocytic hemolytic anemia is a rare condition with an autosomal recessive pattern. It is due to altered RBC metabolisms and shows the absence of any spherocytes or inclusion bodies on peripheral smear [1]. Clinical symptoms include pallor and fatigue from anemia as well as dark yellow urine and jaundice from hyperbilirubinemia. Patients are usually negative for any autohemolytic tests such as Coombs test. Our patient was a previously healthy female, with an apparent family history of similar complaints from her mother and maternal aunt. Her peripheral smear also was compliant with the diagnosis of CNSHA. Patients with CNSHA tend to have a normal hemoglobin structure and stability. Different enzymes deficiencies have been reported such as pyruvate kinase deficiency, hexokinase deficiency [2], pyrimidine 5’nucleotidase deficiency [3] and homozygous glucose phosphate isomerase (GPI) deficiency [4] causing a decrease in adenosine triphosphate (ATP) levels which eventually leads to RBC death and hemolysis [5-6]. Warang et al. used a molecular modeling to show that mutations of L487F can cause a loss of the ability of GPI to dimerize, causing the RBCs to have lower thermostability and significant changes in metabolisms leading to hemolysis [7].

Treatment of the condition involves symptomatic care and the patient should be advised to avoid any factors that can precipitate the hemolysis such as any drugs, stress or food [8]. Recovery of our patient was uneventful following the proper management of her anemia and symptoms. She was also advised to seek genetic testing and counseling before any planned pregnancies.

## Conclusions

Congenital non-spherocytic hemolytic anemia is a rare autosomal recessive condition causing enzyme deficiencies that lead to congenital hemolytic anemia and usually no spherocytosis and hemoglobin abnormalities are present. Clinical symptoms usually include jaundice from high levels of bilirubin and pallor and fatigue from anemia. Treatment consists of symptomatic management and proper advice on genetic counseling.
